# Iberdomide (CC-220) is a potent cereblon E3 ligase modulator with antitumor and immunostimulatory activities in lenalidomide- and pomalidomide-resistant multiple myeloma cells with dysregulated *CRBN*

**DOI:** 10.1038/s41375-019-0620-8

**Published:** 2019-11-12

**Authors:** Chad C. Bjorklund, Jian Kang, Michael Amatangelo, Ann Polonskaia, Mark Katz, Hsiling Chiu, Suzana Couto, Maria Wang, Yan Ren, Maria Ortiz, Fadi Towfic, J. Erin Flynt, William Pierceall, Anjan Thakurta

**Affiliations:** 1 Celgene Corporation, Summit, NJ USA; 2 Celgene Corporation, San Diego, CA USA; 3 Celgene Corporation, CITRE, Sevilla, Spain

**Keywords:** Cancer therapeutic resistance, Myeloma

## To the Editor:

Immunomodulatory compounds, IMiDs® lenalidomide and pomalidomide mediate their anti-myeloma activities via cereblon, a component of the Cul4A^*CRBN*^ E3 ligase complex [1, 2]. Here we describe preclinical experiments with a next generation cereblon targeting agent, iberdomide (CC-220) and explore its activity in lenalidomide- and pomalidomide-sensitive and -resistant multiple myeloma (MM) cell lines, and its pharmacodynamic effects in the bone marrow from relapsed/refractory MM (RRMM) patients.

We observed order of antiproliferative activity of iberdomide > pomalidomide > lenalidomide in matched lenalidomide-sensitive (H929) and an acquired lenalidomide-resistant (H929/LR) cell line (Fig. S1A). In a panel of MM cell lines across a range of concentrations, iberdomide had pronounced antiproliferative effects (Fig. S1B) compared to lenalidomide and pomalidomide measured by a ‘sensitivity shift’ of the relative percentage of AUC reduction (Supplementary Methods) (Fig. S1C). Analysis of the substrates Aiolos/Ikaros show degradation by both pomalidomide and iberdomide in the H929/LR cells (not shown), consistent with previous observations [3]. Further, treatment of H929 cells, with either pomalidomide or iberdomide resulted in time-dependent increases in G0/G1 and sub-G1 cell cycle fractions (Fig. S1D). Consistently, iberdomide induced greater apoptosis than pomalidomide in all MM cell lines tested, at a tenfold lower concentration, estimated to be in the range of clinical activity [4] (Fig. S1E).

Pomalidomide and lenalidomide bind cereblon with similar affinity (~3 µM) [5]. We previously reported that faster rate of degradation of targeted substrates, Ikaros and Aiolos, and the down regulation of c-Myc/IRF4 expression were associated with greater antitumor effects of pomalidomide [6]. Treatment with 0.1 µM iberdomide led to a faster decrease in the relative abundance of these proteins than with pomalidomide (1 µM) (Fig. S1F). Cereblon-binding affinity IC50 of iberdomide is ~150 nM [5]. Thus the faster degradation of the substrates may be due to increased cereblon-binding affinity and/or improved processivity of the iberdomide-bound E3 ligase.

Current clinical application of IMiDs compounds include doublet and triplet combinations with dexamethasone, bortezomib, and/or daratumumab. We initially compared the antiproliferative and pro-apoptotic activity of iberdomide to pomalidomide in combination with bortezomib in MM1.S cells. Due to the potent cytotoxic effects of bortezomib, pomalidomide, and iberdomide, and the narrow window of observable combinatorial effects, we titrated either pomalidomide (0.001–10 μM) or iberdomide (0.0001–1 μM) against bortezomib (0.0625–1 nM) (Figs. S2A, S3A, left). Using these concentrations, inhibition of proliferation induced by the combinations of iberdomide/bortezomib and pomalidomide/bortezomib were both synergistic [7] (Fig. S2B). In MM1.S cells, while single agent bortezomib, pomalidomide, or iberdomide induced apoptosis at 11%, 77%, and 89% respectively, the combination of iberdomide/bortezomib increased the apoptotic fraction to 95%, compared to pomalidomide/bortezomib at 87% (Fig. S2C). Utilizing similar concentrations where we observed synergy with bortezomib, we evaluated the potential inhibitory effect on substrate degradation of Aiolos, Ikaros, and ZFP91, and found no apparent inhibition by bortezomib with either pomalidomide or iberdomide (Fig. S2D).

While the combination of iberdomide with bortezomib displayed strong antitumor effects, a potential clinical combination would likely include dexamethasone. Proliferative inhibition in MM1.S cells with the combination of iberdomide/bortezomib (Fig. S3A, left), followed by the addition of 1 nM dexamethasone increased the sensitivity (Fig. S3A, middle), and addition of 10 nM dexamethasone nearly completely stopped all proliferation (Fig. S3A, right). Combination index calculations [7] showed a synergistic antiproliferative effect across the concentration range for the three drugs (Fig. S3B).

In presence of human-derived complement, iberdomide plus daratumumab had a greater inhibitory effect on H929 cells than either drug alone (Fig. S3C). While complement-dependent cytotoxicity (CDC) was reported to be the primary mechanism of action for daratumumab, it also exerts activity through antibody-derived cellular cytotoxicity (ADCC) [8]. We evaluated the effects of iberdomide and daratumumab, alone and in combination in an ADCC assay. First, we incubated isolated PBMCs (effector) with either vehicle (DMSO), daratumumab (Dara (0.1 μg/mL)), iberdomide (Iber (0.008 μM)), or both drugs (Fig. S3D), and measured ADCC on the target H929 cells (Supplemental Methods). H929 only, PBMCs alone and PBMCs treated with daratumumab had similar killing effects on the target cells (purple and blue bars), while iberdomide (green) and iberdomide/daratumumab (red) had more cell killing activity (Fig. S3D; left group of bars; H929). Next, we treated the effector PBMCs as before, but additionally treated the target cells with daratumumab (Fig. S3D; second group of bars; H929 + dara). This resulted in an increased PBMC-mediated killing with PBMCs alone (purple), with daratumumab (blue), and a more pronounced effect with iberdomide (green) or iberdomide/daratumumab (red). We tested additional combinations, including the target treated with only iberdomide (Fig. S3D; H929 + iber) or with both drugs (Fig. S3D; H929 + dara + iber), and as expected the ADCC killing effects were greater with each addition. These results highlight the potent immune-mediated cytotoxicity of iberdomide alone and its ability to augment daratumumab mediated ADCC presumably by stimulation of NK cells and thus counteracting the latter’s known NK–NK cell fratricidal killing effects [8].

In order to study the activity of iberdomide in a pomalidomide-resistant setting, we generated a panel of pomalidomide-resistant (PR) cell lines (*n* = 9) using methods previously described [9]. Overall, there was a general trend of reduced cereblon protein and mRNA expression in nearly all PR cell lines relative to parental sensitive (S) cell lines (Figs. S4A, S4B, and Table S1); however, cereblon was clearly detectable in several cell lines. The greatest reduction was seen in the H929/PR cells (88% reduction), compared to the previously published DF15/PR [1] cells (97% reduction) (Fig. S4A; Table S1). We also analyzed *CRBN* gene mutation status in the cell lines by NGS. Interestingly, in three cell lines there were alterations in the *CRBN* gene (Table S1). The EJM/PR line had an intronic SNV and H929 had two mutations that resulted in both an insertion and a deletion. The MM1.S/PR cell line was unique as it contained a 12-base pair intronic deletion, resulting in a *CRBN* transcript with a subsequent deletion of exon 6 of *CRBN* (*CRBN∆6*). The *CRBN∆6* protein product was detectable by western running at a slightly smaller molecular weight (Fig. S4A).

**Fig. 1:**
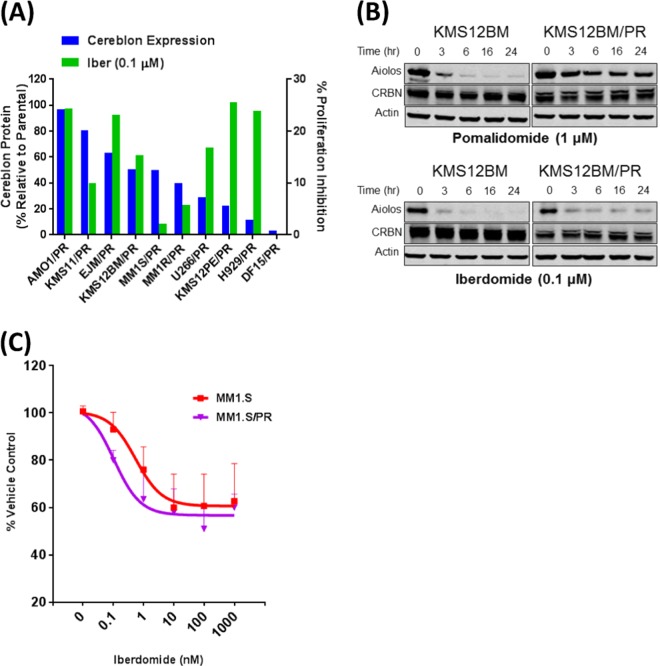
Iberdomide activity in pomalidomide-resistant cell lines. **a** Relative cereblon protein (blue bars; left *Y*-axis) in the pomalidomide-resistant cell lines as determined by densitometry of the Western blots from Supplemental Fig. 3A, normalized to their individual actin loading control and to their parental sensitive counterparts. Represented as % change relative to parental. Overlaid is the relative % proliferation inhibition of iberdomide (iber – 0.1 μM; green bars) on those individual cell lines as determined by 3H-thymidine incorporation. **b** Western blot analysis showing the effects of either pomalidomide (1 μM) or iberdomide (0.1 μM) on the degradation kinetics of Aiolos in the parental sensitive KMS12BM and pomalidomide-resistant KMS12BM/PR. Actin is shown as a loading control. **c** PBMC co-culture experiments where isolated, CD3-stimulated PBMCs were incubated with iberdomide (0.0001–1 μM) for 72 h, and then combined with either the parental MM1.S (CFSE-stained) or pomalidomide-resistant MM1.S/PR (CFSE-stained) cells for the final 4 h. CFSE + cells were gated on and evaluated for apoptosis by flow cytometry using Annexin-V and ToPro3^+^ staining. Bars represent % of viable target cells compared to vehicle control (DMSO). Shown here is the representative of three independent experiments

Next, we tested iberdomide activity in PR cell lines with respect to cereblon levels and mutations. To do that, relative cereblon protein expression levels in the PR lines were compared to the isogenic sensitive parental lines as determined by western and densitometry (100% representing no change) (Fig. 1a; left *Y*-axis, blue bars and Fig. S4A). Overlaid on top of this graphic is the % inhibition of proliferation by iberdomide (0.1 μM) for each PR cell line (right *Y*-axis, green bars). In seven of the ten PR cell lines where cereblon expression was reduced but detectable (AMO1/PR, KMS11/PR, KMS12BM/PR, U266/PR, and KMS12PE/PR) (Fig. S4A), there was the modest antiproliferative activity of iberdomide. In contrast, there was very little if any measurable activity in the MM1.R/PR, MM1.S/PR, or the DF15/PR lines. Interestingly, there was no obvious correlation between cereblon level and iberdomide’s anti-proliferative activity. In addition, iberdomide displayed some anti-proliferative activity in two of the PR lines with cereblon mutations (EJM/PR and H929/PR, Table S1) along with decreased levels of cereblon protein. NGS analysis of the PR lines did not reveal other mutations in the Cul4A^*CRBN*^ E3 ligase, substrates or downstream pathway(s) (data not shown).

To gain insight into the potential mechanisms of iberdomide in PR cells, we analyzed Aiolos degradation in KMS12BM and KMS12BM/PR lines treated with either pomalidomide or iberdomide. As expected, both pomalidomide and iberdomide led to rapid Aiolos depletion in the KMS12BM line (Fig. 1b). In contrast, only iberdomide was effective at inducing rapid Aiolos depletion in the KMS12BM/PR cells. We observe similar results with Ikaros (data not shown).

To evaluate the immunomodulatory effects of iberdomide in PR cells, we performed PBMC co-culture killing experiments. Iberdomide equally induced PBMC-mediated killing of both parental MM1.S cells and MM1.S/PR cells (Fig. 1c). This is important because there was no direct cytotoxic activity of iberdomide on MM1.S/PR. Next, we evaluated the combinations of iberdomide in PR cell lines with daratumumab. First, iberdomide was combined with daratumumab for CDC in the H929/PR cell line. In the presence of human-derived complement, iberdomide/daratumumab combination had a more pronounced dose-dependent inhibitory effect on H929/PR cells than either drug alone (Fig. S4C). In addition, the combination of iberdomide and bortezomib, in KMS12PE/PR cells demonstrated an enhanced anti-proliferative effect at a low concentration of bortezomib (0.25 nM) (Fig. S4D).

**Fig. 2:**
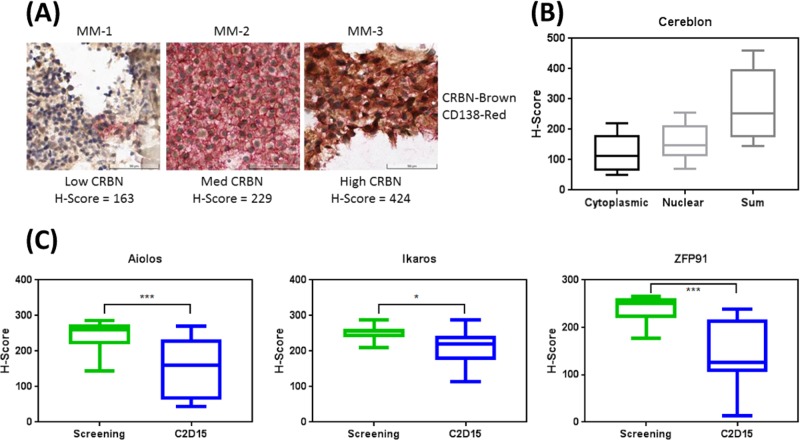
Wide range of cereblon protein expression in the bone marrow of MM patients previously treated with IMiDs. **a** Dual stained (CD138^+^ = red and cereblon = brown) immunohistochemical analysis of bone marrow tissue at screening for ten patients in the ongoing clinical study (NCT02773030). **b** Compiled H-score boxplot analysis (see “Materials and methods”) comparing nuclear, cytoplasmic and total cereblon staining in IHC samples shown in **a**. **c** Compiled IHC *H*-score analysis of ten patients of either Ikaros, Aiolos or ZFP91 on screening and at cycle 2 day 15

Currently, a phase 1b/2a study (clinicaltrials.gov #NCT027730300) is ongoing to determine the maximum tolerated dose of iberdomide alone or in combination with dexamethasone in RRMM. For exploratory analysis, bone marrow aspirate clots were collected at both baseline and on cycle 2 day 15 (C2D15) and analyzed by immunohistochemistry (IHC) [10]. There was a dynamic range of cereblon expression at screening, from low to high (Fig. 2a), and a wide range of both cytoplasmic, nuclear, and total expression (*n* = 10; 9 of 10 were refractory to pomalidomide) (Fig. 2b), consistent with the acquired PR cell lines (Fig. S4A). The pharmacodynamic effects of iberdomide was assessed by comparing screening and on treatment (C2D15) bone marrow samples for Ikaros, Aiolos, and ZFP91 (Fig. S5A). Cumulative *H*-score assessment (*n* = 10) showed significant decreases in substrate proteins during iberdomide therapy (Fig. 2c). These results therefore recapitulated iberdomide’s biochemical activity in patient MM cells demonstrating efficient pharmacodynamic effects.

Overall, iberdomide biochemical potency translates into greater anti-MM activity than lenalidomide or pomalidomide in both IMiD-sensitive and -resistant MM cell lines (Fig. S5B). These results provide strong preclinical and translational evidence for iberdomide activity and its potential for clinical development in MM in combination with other agents, especially with bortezomib and daratumumab in RRMM.

## Supplementary information

Supplemental Methods and Figures
