# Supplementary material for: Iberdomide (CC-220) is a potent cereblon E3 ligase modulator with antitumor and immunostimulatory activities in lenalidomide- and pomalidomide-resistant multiple myeloma cells with dysregulated CRBN
Source: Leukemia. 2019 Nov 12;34(4):1197–201. doi: 10.1038/s41375-019-0620-8 (PMC7214241; doi:10.1038/s41375-019-0620-8)
Supplement: Supplementary file 1 — Supplemental Methods and Figures [file 41375_2019_620_MOESM1_ESM.pdf]

Supplemental Methods and Figures:

Cell Lines

Human MM-derived cell lines (ATCC, Manassas, VA, USA, DSMZ, Braunschweig, Germany. or JCRB, Ibaraki City, Osaka, Japan) were maintained and routinely tested for mycoplasma and authenticated by STR fingerprinting (ThermoFisher, AmpFlSTR) as previously described.(1, 2) Acquired pomalidomide-resistant cell lines(2) were maintained in the presence of 10 μM pomalidomide unless used for experiments in which pomalidomide was removed from culture for at least 7 days prior.

Reagents

Bortezomib and dexamethasone were obtained from Selleck Chemicals (Houston, TX, USA). Daratumumab was from BlueDoor Pharma (Rockville, MD, USA).

Proliferation Assays

Cellular proliferation was assessed using ^{3}H-thymidine incorporation assay as previously described.(1) Briefly, the MM lines were cultured in triplicate or quadruplicate wells in 96-well plates with indicated concentrations of drugs for 72-96 hrs (as indicated per experiment) and ^{3}H-thymidine was added to the culture medium for the final 6 hours. ^{3}H-thymidine incorporation was determined in a liquid scintillation counter and data collected and analyzed.(1)

Calculation of AUC Reduction

For analysis and comparison of different compounds and effect on proliferation over similar concentration ranges, we chose not to use EC50 or IC50 comparisons as these measurements were accurately representative of differential activity due to the shapes of the fitted curves and levels E_{max}. Instead we opted for Comparison of the area under the curve (AUC), and the relative AUC reduction percentage $((1-(AUC^{iberdomide}/AUC^{pomalidomide}))*100)$, represents the relative 'sensitivity shift'.

Calculation of Synergy

The synergy of drug combinations was analyzed by using the Chou-Talalay(3) using Calcusyn (Biosoft, Great Shelford, Cambridge, UK). The combination index (CI) was defined as a

quantitative measure of the degree of drug interaction in terms of additive effect (CI=1), synergism (CI<1), or antagonism (CI>1) for a given endpoint of the effective measurement.

Apoptosis and Cell Cycle Analysis by Flow Cytometry

Apoptosis was measured by To-Pro-3 and Annexin V incorporation.(1) Cell cycle analysis was measured by propidium iodide staining.(4)

Immunoblotting

Cells for immunoblotting were prepared(1) and probed with respective primary antibodies overnight, followed by incubation with IR-dye secondary antibodies (LI-COR Biotechnology, Lincoln, NE, USA). Protein bands were visualized (1) followed by densitometric quantification and analysis using Image Studio Software (LI-COR). Protein density was normalized to β-actin loading controls and parental controls as indicated, arbitrarly set at 100%. The percentage of reduced cereblon expression was calculated by subtracting the relative percentage of expression of resistant cell line from 100% of parental cell line. The following antibodies were used for immunoblotting: Ikaros (Santa Cruz Biotechnology, Dallas, TX, USA), Aiolos (Santa Cruz and Abcam, Cambridge, MA, USA), IRF4 (Santa Cruz), c-Myc (Abcam), cereblon (Celgene; CELG-4-6-5), ZFP91 (LS-Bio, Seattle, WA, USA), cleaved PARP-1 (Cell Signaling Technology, Danvers, MA, USA), and β-actin (LI-COR).

Peripheral Blood Mononuclear (PBMC) Co-culture with MM Cells

Immune-stimulated PBMC co-culture killing assay was performed as previously described.(5) Peripheral blood mononuclear cells (PBMCs) were obtained from healthy untreated donors by Ficoll gradient. Anti-CD3 (OKT3; eBiosciences, Springfield, NJ, USA) stimulated PBMCs were cultured with iberdomide (0.0001-1 μM) for 72 hours and culture supernatants for IL-2 were assessed using ELISA. PBMCs were washed to remove residual compound, andsubsequently co-cultured with carboxyfluorescein succinimidyl ester (CFSE; ThermoFisher, Waltham, MA, USA)-labelled MM cells at a 3:1 ratio for 4 hrs. The culture supernatants were collected and assayed for Granzyme B secretion by ELISA. Co-cultured cells were washed and stained with Annexin-V-PE and To-Pro3-APC for apoptosis measurement on CFSE-labelled MM cells.(6)

CDC Assays

For complement-dependent cytotoxicity (CDC) assay, target cells were treated with the indicated drug (iberdomide or daratumumab) with and without the presence of 15% human serum (Sigma-Aldrich; St, Louis, MO, USA) containing complement. Heat inactivated serum was also utilized as a control. Cells were plated in triplicate or quadruplicate wells in 96-well plates with the indicated drug for 72 hrs, followed by ^{3}H-thymidine addition to the culture medium for the final 6 hours. The ^{3}H-thymidine incorporation was determined in a liquid scintillation counter.(1) All data was normalized to either vehicle or isotype control.

ADCC Assay

Isolated, CD3-stimulated PBMCs (effector cells), H929 MM cells (target), or both were treated with either vehicle (DMSO), daratumumab (dara (0.1 µg/mL)), iberdomide (0.008 µM) (iber), or both drugs (Figure 2C), and measured ADCC on the target H929 cells. Following washout of drug (iberdomide or daratumumab at the indicated concentrations) on either target cells or PBMCs, target cells were labeled with CSFE and then combined with PBMCs at a 3:1 ratio for 4 hrs. Co-cultured cells were washed and stained with Annexin-V-PE and To-Pro3-APC and target cells were then gated by CFSE and measured for apoptosis.

IL-2 and Granzyme B ELISA

Supernatants from co-culture experiments were analyzed for IL-2 production and Granzyme B release using ELISA kits and according to following manufacturer's protocol (R&D and Biolegend, respectively).

Quantitative RT-PCR (qRT-PCR)

Total RNA was isolated from harvested cell pellets using RLT Buffer (Qiagen; Germantown, MD, USA) per manufacturer's directions. cDNA was synthesized using a cDNA Reverse Transcription kit (Applied Biosystems; Foster City, CA, USA) according to the manufacturer's protocol. qRT-PCR was performed using the TaqMan Gene Expression Master Mix and TaqMan Gene Expression Assays as triplicate/quadruplicate samples on a Viaa7 PCR System (Applied Biosystems). Relative quantification was assessed by comparative CT method after normalization to internal GAPDH control;all samples were then normalized to either parental or vehicle controls (arbitrarily set to 1).

Immunohistochemistry

Primary MM bone marrow samples from patients in the Phase 1b/2a study CC-220-MM-001 (clinicaltrials.gov trial # NCT02773030) were dual stained with either CD138/Cereblon, CD138/Ikaros, CD138/Aiolos or CD138/ZFP91 were applied using the Leica Bond automated slide stainer.(7) Anti-CD138 mouse monoclonal antibody (Dako North America, Inc, Carpenteria, CA, USA) was used at a 1/1200 dilution and was detected using Bond Polymer Refine Red Detection kit. Cereblon, Aiolos, Ikaros and ZFP91 were detected using the antibodies Cereblon (rabbit monoclonal; CELG645), Aiolos (rabbit monoclonal; CELG977), Ikaros (rabbit polyclonal antibody; Millipore, Billerica, MA), ZFP91 (rabbit monoclonal; Abcam), c-Myc (rabbit monoclonal; Abcam)), and IRF4 (rabbit polyclonal antibody; Millipore, Billerica, MA). IHC immunoreactivity intensity was scored on a scale of 0-3 (0 = negative, 1 = weak, 2 = intermediate, 3 = strong). Percentages of cells with each intensity were recorded. H-score was calculated as the sum of the intensity multiplied by the percentage of positively staining tumor cells.

NGS of *CRBN* in Pomalidomide-Resistant MM Cell Lines

We generated a set of pomalidomide-resistant cell lines (n=9).(2) Following generation of these cell lines, we characterized *CRBN* gene status by next generation sequencing (NGS) in both the parental and resistant pairs. Genomic DNA was extracted from frozen cell pellets and library preparation was performed (Cancer Genetics Inc., Rutherford, NJ) with a targeted hybrid-capture procedure using a custom probe panel (Nimblegen) comprising the coding regions of the selected genes. Individual libraries were prepared from sheared DNA using the KAPA Hyper Library Preparation kit (KAPA Biosystems) and barcoded, prior to multiplexing and hybridization. After DNA recovery, libraries were submitted to bi-directional sequencing on a Nextseq500 system (Illumina). De-multiplexed paired sequencing reads were aligned using BWA-MEM with human genome build GRCh37/hg19 as reference, followed by variant calling using the Varscan variant caller algorithm. Variant annotation was performed using Cartegenia Bench Lab NGS v4.2.2 (Agilent). Variant filtering was done using Agilent's Bench workstation using custom filter (synonymous variants, variants outside of +/-2bp of exonic boundaries and population frequency of >1% were excluded from analysis). For non-truncating variants, functional impact was assessed primarily using in silico algorithms for protein prediction.

Figure S1

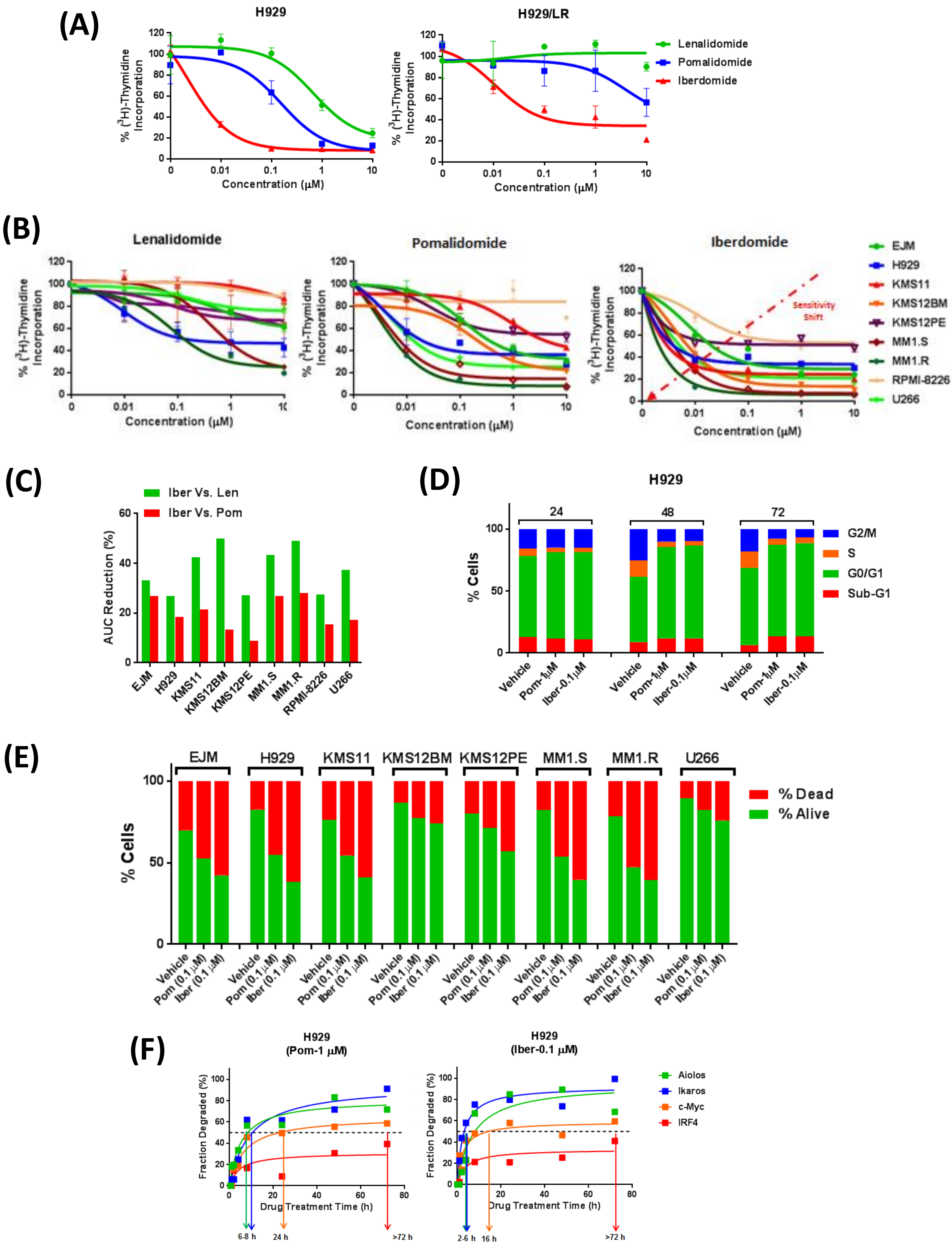
(A)
H929
H929/LR
% (3H)-Thymidine Incorporation
Concentration (µM)
Lenalidomide
Pomalidomide
Iberdomide
(B)
Lenalidomide
Pomalidomide
Iberdomide
Sensitivity Shift
EJM
H929
KMS11
KMS12BM
KMS12PE
MM1.S
MM1.R
RPMI-8226
U266
(C)
Iber Vs. Len
Iber Vs. Pom
AUC Reduction (%)
(D)
H929
24
48
72
% Cells
G2/M
S
G0/G1
Sub-G1
Vehicle
Pom-1µM
Iber-0.1µM
(E)
EJM
H929
KMS11
KMS12BM
KMS12PE
MM1.S
MM1.R
U266
% Cells
% Dead
% Alive
Vehicle
Pom (0.1 µM)
Iber (0.1 µM)
(F)
H929 (Pom-1 µM)
H929 (Iber-0.1 µM)
Fraction Degraded (%)
Drug Treatment Time (h)
Aiolos
Ikaros
c-Myc
IRF4
6-8 h
24 h
>72 h
2-6 h
16 h
>72 h

Figure S1: Iberdomide is more potent than pomalidomide against MM cell lines in vitro. (A) The anti-proliferative activity of iberdomide was directly compared to lenalidomide and pomalidomide in lenalidomide-sensitive H929 and lenalidomide-resistant H929/LR cell lines across a range of concentrations (0.01-10 μM) for 72 hrs as measured by ^{3}H-thymidine incorporation. **(B)** The anti-proliferative activity of iberdomide was directly compared to lenalidomide and pomalidomide in a panel of MM cell lines similar to that in **(A)**. **(C)** The area under the curve (AUC) was calculated for either pomalidomide or iberdomide growth curves and the normalized % AUC reduction $((1-(AUC^{iberdomide}/AUC^{pomalidomide}))*100)$ were plotted as a measure to represent the relative 'sensitivity shift' shown in part **(B)**. **(D)** Cell cycle analysis as measured by flow cytometry and PI staining staining following either vehicle (DMSO), 1 μM pomalidomide (Pom) or 0.1 μM iberdomide (Iber) on H929 cells after 24, 48 or 72 hrs. (**E)** Apoptosis measured by flow cytometry using Annexin-V ($AnnV^{+}$; x-axis) and $ToPro3^{+}$ (y-axis) staining following either vehicle (DMSO), 1 μM pomalidomide (Pom) or 0.1 μM iberdomide (Iber) on H929 cells following 96 hrs. **(F)** Kinetic analysis measuring the relative protein abundance of Aiolos, Ikaros, c-Myc and IRF4 in the H929 cell line following treatment with either 1 μM pomalidomide (Pom) or 0.1 μM iberdomide (Iber). Figures shown are representatives of at least n = 3 experiments.

Figure S2

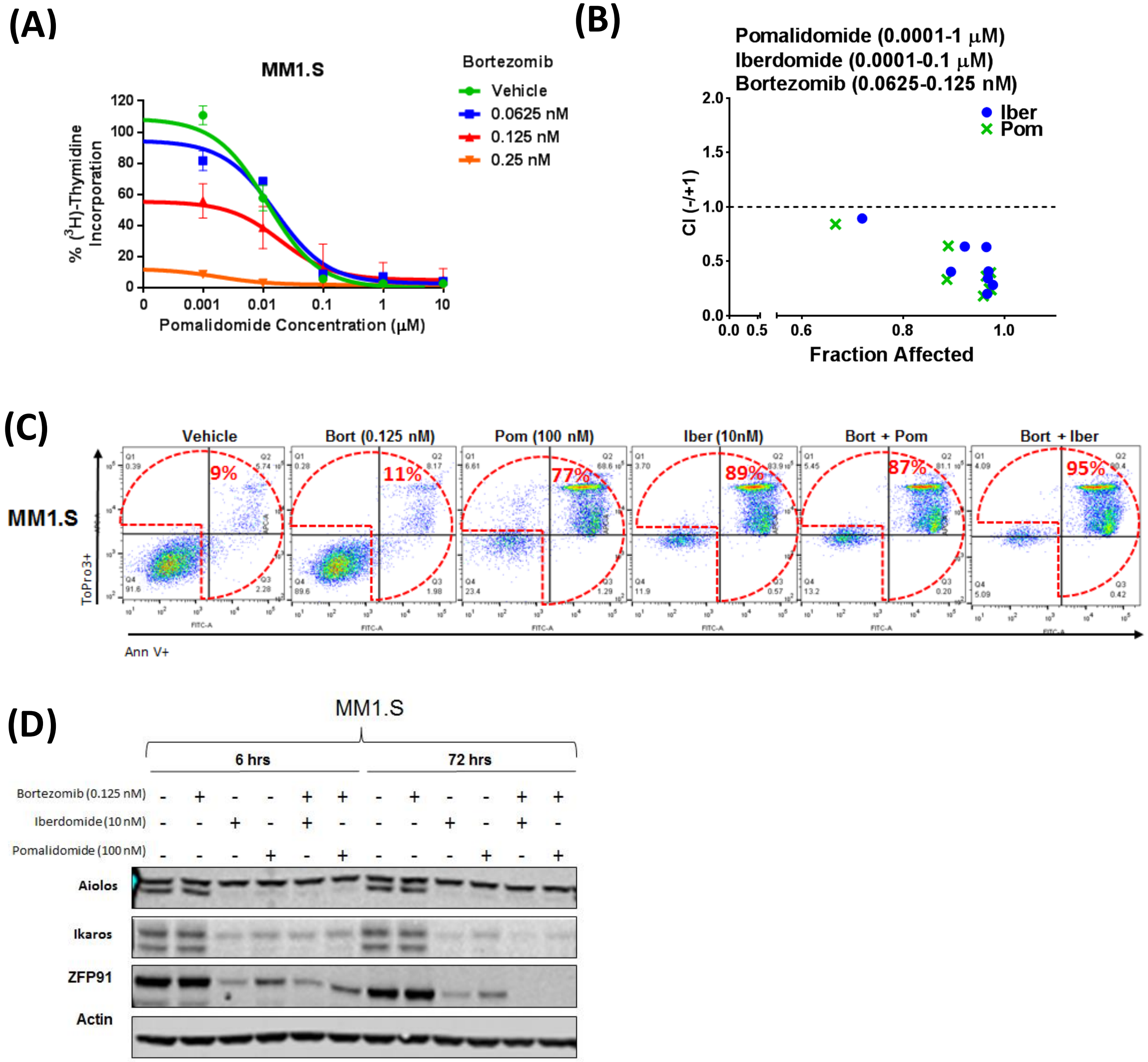

Figure S2: Iberdomide shows better combinatorial activity with bortezomib than pomalidomide. (A) The anti-proliferative activity of pomalidomide (0.001-1 μM) combined with bortezomib (0.0625, 0.125, and 0.25) as measured by ^{3}H-thymidine incorporation in the sensitive MM1.S cell line. **(B)** Combination indices calculated for the combination of bortezomib (0.01-0.25 nM) with either pomalidomide (0.001-1 μM) or iberdomide (0.001-1 μM). **(C)** Apoptosis measured by flow cytometry using Annexin-V (AnnV^{+}; x-axis) and ToPro3^{+} (y-axis) staining following either vehicle (DMSO), 1 μM pomalidomide (Pom) or 0.1 μM iberdomide

(Iber), or in combination with bortezomib (0.125 nM) on MM1.S cell lines following 96 hrs. **(D)** Western blot analysis of cereblon-induced substrates, Aiolos, Ikaros and ZFP91 measured in MM1.S cells following incubation with either vehicle (DMSO), 0.1 μM pomalidomide, 0.01 μM iberdomide, or in combination with bortezomib (0.125 μM). β-actin is shown as a loading control.

Figure S3

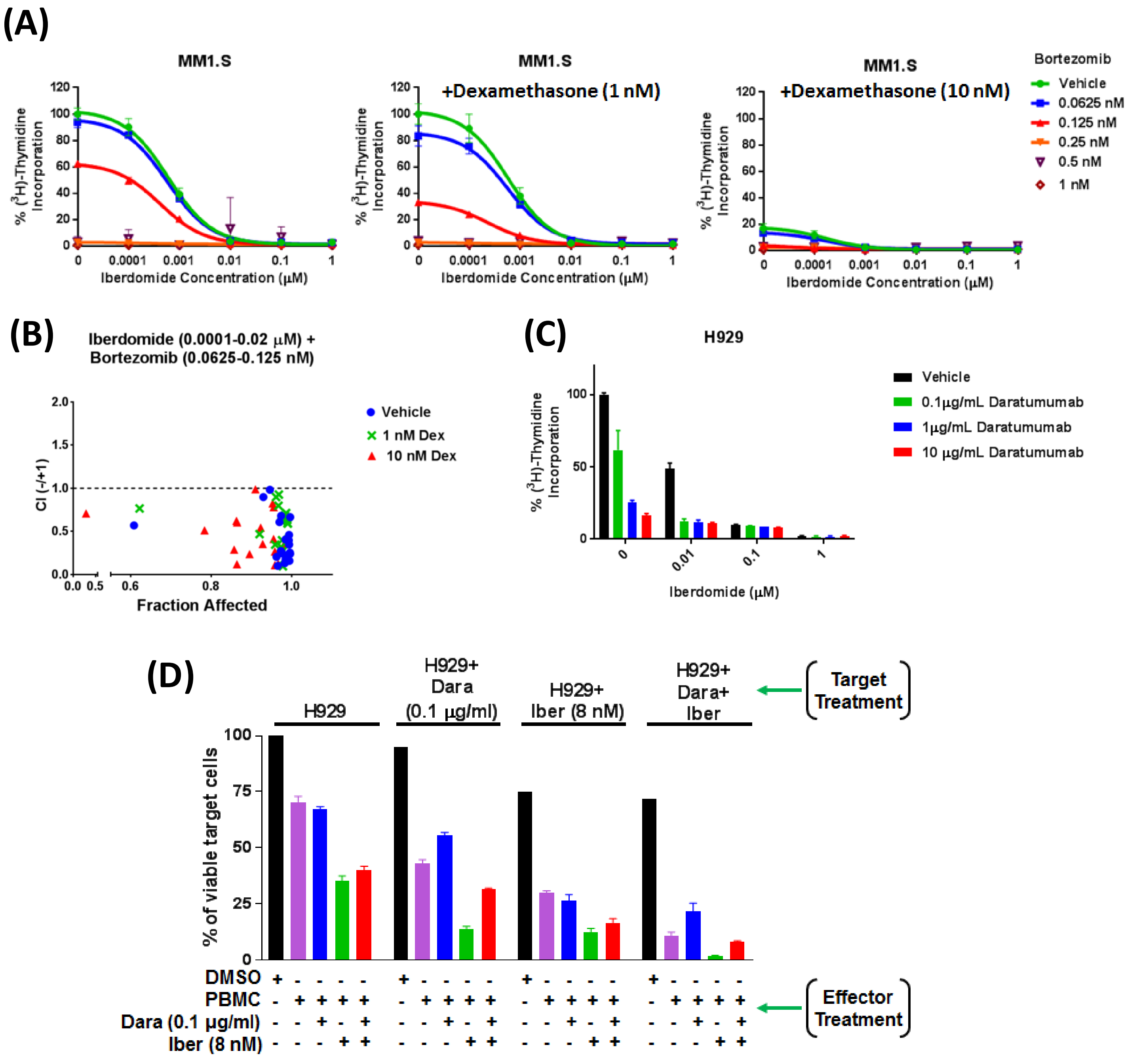

Figure S3: Potential clinical combinations of iberdomide against MM cell lines in vitro. (A) The anti-proliferative activity as measured by ^{3}H-thymidine incorporation of iberdomide (0.0001-1 µM), as a single agent (green line curve) or in combination with bortezomib (0.0625, 0.125, 0.25, 0.5, and 1 nM) are shown in the left panel, or in the presence of dexamethasone (1 nM; middle panel), or (10 nM; right panel). **(B)** Combination indices calculated for the

combination of bortezomib (0.0625-0.125 nM) with iberdomide (0.001-0.02 μM) and dexamethasome (1 nM and 10 nM) as shown in **Figure S3A**. **(C)** Complement-dependent cytotoxicity (CDC), as measured by ^{3}H-thymidine incorporation, following treatment of H929 cells with iberdomide (0.01-1 μM) as a single agent or in combination with daratumumab (0.01-10 μg/mL). **(D)** Treatment of either target (CFSE-labeled H929) cells or PBMCs with either single agent iberdomide (0.008μM), single agent daratumumab (0.1 μg/mL), or in combination for 72 hrs, followed by a four-hour coculture of target cells and PBMCs. CFSE+ cells were gated on and evaluated for apoptosis by flow cytometry using Annexin-V and ToPro3^{+} staining. Bars represent % of viable target cells remaining. Shown here is representative of three independent experiments.

Figure S4

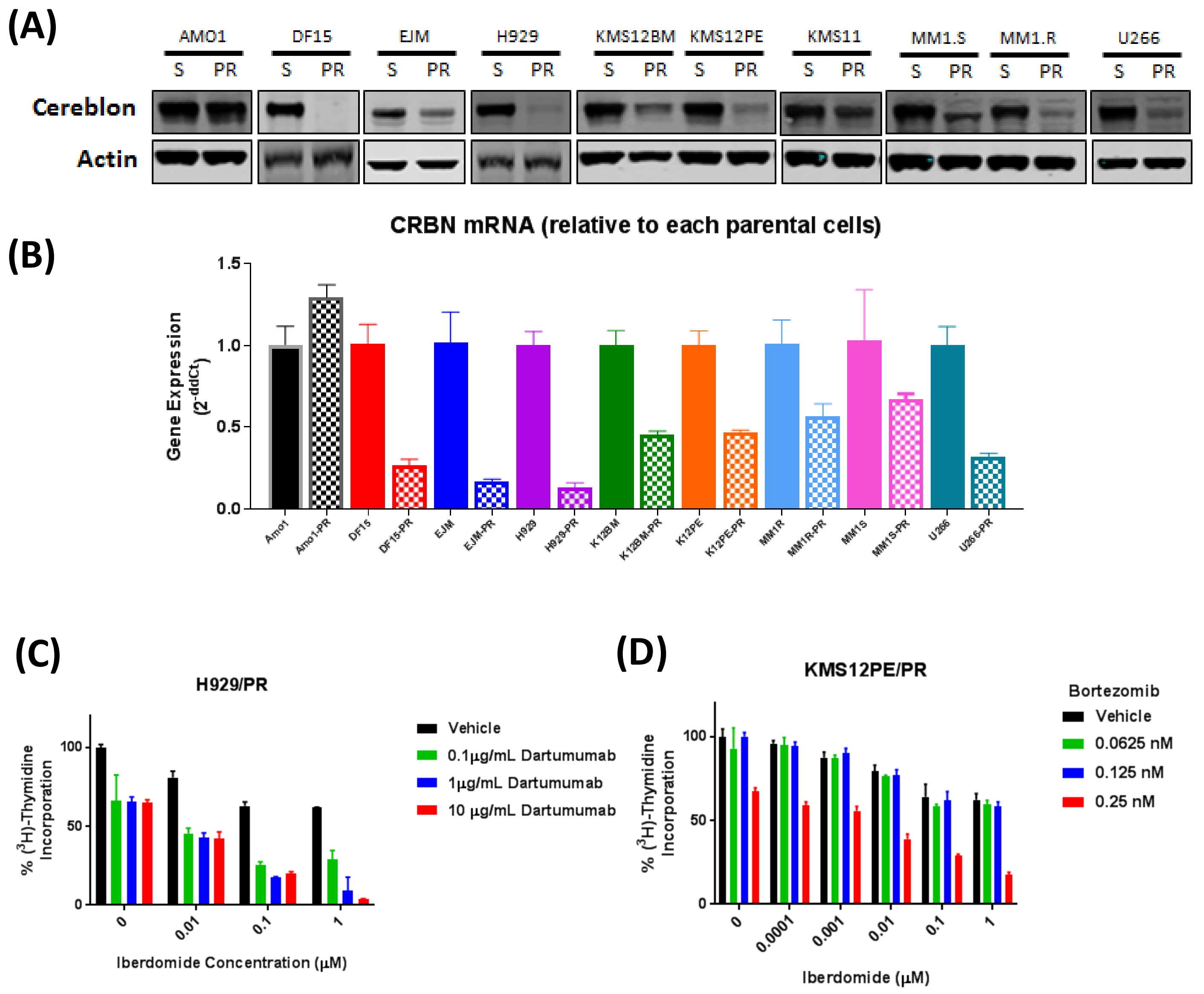

Figure S4: Characterization of pomalidomide-resistant cell lines. (A) Western blot analysis of cereblon expression in parental sensitive (S) cell lines compared to the acquired pomalidomide-resistant (PR) cells generated from a panel of cell lines. **(B)** Real-time PCR showing the relative CRBN mRNA in the parental and pomalidomide-resistant cell lines. The relative amount is normalized to the parental cell line and arbitrarily set at 1. **(C)** Complement-dependent cytotoxicity (CDC), as measured by ^{3}H-thymidine incorporation, following treatment of pomalidomide-resistant H929 cells with iberdomide (0.01-1 µM) as a single agent or in

combination with daratumumab (0.01-10 μg/mL). **(D)** The anti-proliferative activity of iberdomide (0.0001-1 μM),+ bortezomib (0.0625, 0.125, 0.25, 0.5, and 1 nM) as measured by ^{3}H-thymidine incorporation in pomalidomide-resistant KMS12PE/PR.

Figure S5

(A)

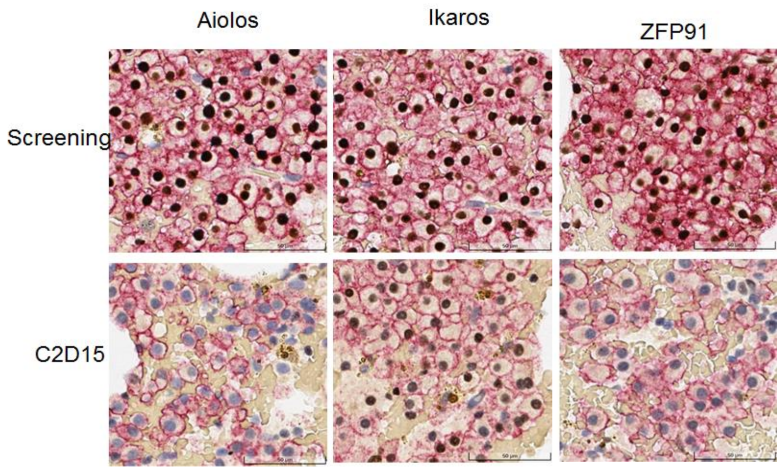

(B)

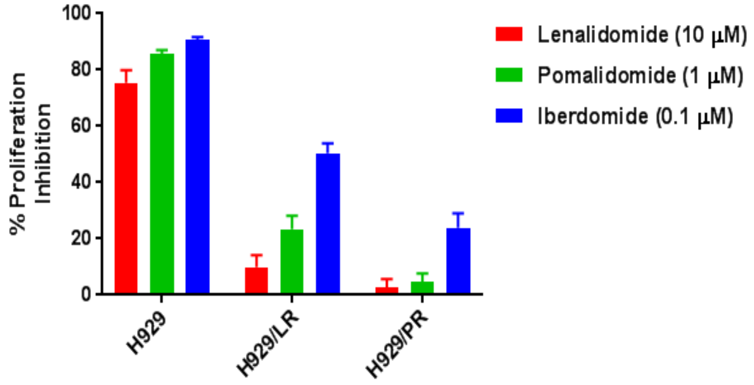

Figure S5: Pharmacodynamic activity of iberdomide on bone marrow samples and different lineages of IMiD resistance. (A) Dual stained ($CD138^{+}$= red and Aiolos, Ikaros, or ZFP91 = brown) immunohistochemical analysis of bone marrow tissue at screening and cycle 2 day 15 (C2D15) on treatment samples. **(B)** The level of proliferation inhibition as measured by ^{3}H-thymidine incorporation in H929 parental, lenalidomide-resistant H929/LR, and pomalidomide-resistant H929/PR when treated with either lenalidomide (10 µM), pomalidomide (1 µM) or iberdomide (0.1 µM).

Table S1

Pom-Resistant Cell Line	% CRBN Transcript by RT-PCR (Relative to Parental)	% Cereblon Protein Expression (Relative to Parental)	CRBN Mutations/Deletions	%anti-proliferative Inhbition with Iberdomide (0.1 µM)
AMO1/PR	↑15%	↑3%	No	31%
DF15/PR	↓74%	↓97%	No	0%
EJM/PR	↓80%	↓37%	Intronic snp (378G>C)	30%
H929/PR	↓82%	↓88%	1. Exonic snp (500T>G; V167G)<br>2. Exonic del (Frameshift 958-967)	24%
KMS12BM/PR	↓55%	↓49%	No	27%
KMS12PE/PR	↓52%	↓78%	No	31%
KMS11/PR	↓44%	↓19%	No	17%
MM1.S/PR	↓32%*	↓49%*	Intronic del Resulting in Exon 6 spliced deletion	4%
MM1.R/PR	↓46%	↓60%	No	6%
U266/PR	↓68%	↓71%	No	22%

Table S1: Characteristics of pomalidomide-resistant (PR) MM cell lines: Relative levels of cereblon mRNA and protein quantified in PR cell lines compared to the parental sensitive lines, including CRBN mutation status, and the relative proliferative inhibition with iberdomide treatment (0.1 µM).

SUPPLEMENTAL REFERENCES:

1. Bjorklund CC, Lu L, Kang J, Hagner PR, Havens CG, Amatangelo M, et al. Rate of CRL4(CRBN) substrate Ikaros and Aiolos degradation underlies differential activity of lenalidomide and pomalidomide in multiple myeloma cells by regulation of c-Myc and IRF4. Blood cancer journal. 2015;5:e354.
2. Bjorklund CC, Ma W, Wang Z-Q, Davis RE, Kuhn DJ, Kornblau SM, et al. Evidence of a Role for Activation of Wnt/β-Catenin Signaling in the Resistance of Plasma Cells to Lenalidomide. Journal of Biological Chemistry. 2011;286(13):11009-20.
3. Rychak E, Mendy D, Shi T, Ning Y, Leisten J, Lu L, et al. Pomalidomide in combination with dexamethasone results in synergistic anti-tumour responses in pre-clinical models of lenalidomide-resistant multiple myeloma. British journal of haematology. 2016;172(6):889-901.
4. Bjorklund CC, Baladandayuthapani V, Lin HY, Jones RJ, Kuiatse I, Wang H, et al. Evidence of a role for CD44 and cell adhesion in mediating resistance to lenalidomide in multiple myeloma: therapeutic implications. Leukemia. 2013.
5. Franssen LE, Nijhof IS, Bjorklund CC, Chiu H, Doorn R, van Velzen J, et al. Lenalidomide combined with low-dose cyclophosphamide and prednisone modulates Ikaros and Aiolos in lymphocytes, resulting in immunostimulatory effects in lenalidomide-refractory multiple myeloma patients. Oncotarget. 2018;9(74):34009-21.
6. Hagner PR, Chiu H, Ortiz M, Apollonio B, Wang M, Couto S, et al. Activity of lenalidomide in mantle cell lymphoma can be explained by NK cell-mediated cytotoxicity. British journal of haematology. 2017;179(3):399-409.
7. Ren Y, Wang M, Couto S, Hansel DE, Miller K, Lopez-Girona A, et al. A Dual Color Immunohistochemistry Assay for Measurement of Cereblon in Multiple Myeloma Patient Samples. Applied immunohistochemistry & molecular morphology : AIMM. 2016;24(10):695-702.
